# Apigenin attenuates serum concentrations of TNF-a, interleukin 1b and interleukin 6 in lipopolysaccharide-stimulated rats 

**DOI:** 10.22038/AJP.2023.23365

**Published:** 2024

**Authors:** Sanaz Jamshidi, Mohammad Sofiabadi, Mina Eslami

**Affiliations:** 1 *Cellular and Molecular Research Center, Research Institute for Prevention of Non-Communicable Disease, Qazvin University of Medical Sciences, Qazvin, Iran *; 2 *Faculty of Hygiene, Qazvin University of Medical Sciences, Qazvin, Iran*

**Keywords:** Inflammation, Apigenin, Dexamethasone, Cytokines

## Abstract

**Objective:**

The use of flavonoids is increasing due to their cost-effectiveness and less adverse reaction. Therefore, the effect of apigenin on lipopolysaccharide (LPS)-induced inflammation was investigated by measuring IL-1b, IL-6, and TNF-a, of serum in the male rats.

**Materials and Methods:**

Ninety male wistar rats were divided in 6 groups included; control, sham, dexamethasone 15 mg/kg, intraperitoneally (i.p.), and apigenin (5, 15, and 30 mg/kg, i.p). Thirty minutes after the administration of solvent or apigenin, LPS (30 μg/kg, i.p) was injected. At time intervals of 4, 12 and 24 hr after injection, blood samples were taken and the concentrations of TNF-a, IL-1b and IL-6 were measured by enzyme-linked immunosorbent assay.

**Results:**

Compared to the control, apigenin (5 mg/kg) decreased the level of TNF-a, and IL-1b in a period of 24 hr (p<0.05). The concentration of IL-6 decreased significantly by apigenin (15 mg/kg) 24 hr after injection (p<0.05). Apigenin (30 mg/kg) decreased the level of TNF-a, at all three time points (4 hr; p<0.05, 12 hr; p<0.01, and 24 hr; p<0.01), and the level of IL-1b (p<0.01), 24 hr and the level of IL-6 at 4 hr (p<0.05), and 24 hr (p<0.01) after LPS injection.

**Conclusion:**

Apigenin can suppress serum inflammatory cytokines, similar to dexamethasone.

## Introduction

An inflammatory response (inflammation) occurs when tissues are injured by pathogens, trauma, toxins, heat, or any other cause (Medzhitov, 2008 ▶). Cytokines are large peptides that are secreted by the immune system. Cytokines are a category of signaling molecules that mediate, and most of them can perform pro- and anti-inflammatory roles. Numerous adverse stimuli such as reactive oxygen species, microbial toxins, and lipopolysaccharide indorse cytokine expression (Han et al., 1998 ▶). Interleukin-1, and tumor necrosis factor alpha (TNF- a) are the main pro-inflammatory cytokines (Nordgreen et al., 2018 ▶). An increase in their level has been reported in most inflammatory diseases such as chronic ulcerative colitis, rheumatoid arthritis, diabetes, arteriosclerosis, some types of cancer and neurodegenerative disorders (Chung et al., 2009 ▶; Holmes, 2013 ▶). Interleukin-6 (IL-6) is a cytokine with extensive pro-inflammatory and anti-inflammatory roles (Pedersen et al., 2008 ▶). IL-6 increasement in the rat brain has been observed after central or peripheral lipopolysaccharide administration (Beurel et al., 2009 ▶). It is also claimed that the inflammatory response to LPS exposure in airway epithelial cells and macrophages is mainly caused by the increase of IL-6, and TNF-a (Liu et al., 2016 ▶).  Sepsis has been shown to increase serum IL-6, and one of the ways to induce experimental sepsis is to administer LPS. IL-6 can have beneficial or harmful effects depending on the amount and continuation of stimulus (Beurel and Jope, 2009 ▶). Foremost anti-inflammatory agents bring various complication, including systemic immunosuppression, and gastrointestinal problems. Therefore, according to these complications, the use of alternative substances has found a special place (Shojaei et al., 2023 ▶). In this regard, the anti-inflammatory effect of flavonoids has been described moreover to the anti-cancer, antioxidant, and antibacterial effects (Babu et al., 2011 ▶; Arun et al., 2016 ▶). Flavonoids have anti-inflammatory activity through several mechanisms, such as reducing transcription factors, and inhibiting some regulatory enzymes that adjust the mediators involved in inflammation (Ribeiro et al., 2015 ▶). One of the important flavonoids is apigenin which is found in celery, chamomile and parsley, and its anti-inflammatory and antioxidant possessions have been confirmed in some studies (Sung et al., 2016 ▶; Leyva-Lopez et al., 2016 ▶). However, all the details of the anti-inflammatory outcome of apigenin have not been properly identified yet. In the present study, the anti-inflammatory effect of apigenin was evaluated by measuring the level of inflammatory cytokines IL-1b, IL-6 and TNF-a, after LPS injection in animals.

## Materials and Methods

**Preparation of animal and materials**

Ninety male rats (Wistar) were purchased from Razi Company (Karaj-Iran) for 200-230 g. Animals were kept 12–12 hours dark/light, temperature 23±1°C and free access to water and food. This research was approved by the ethics committee of research center (IR.QUMS.REC.1402.008). ELISA kits (Oscan-USA), and other materials were prepared from Sigma-Aldrich (Germany).

**Main intervention**

Animals were randomly divided into six equal groups of control (LPS, without mediation), sham (solvent; normal saline), experimental groups of apigenin (5, 15 and 30 mg/kg) and dexamethasone (positive control, 15 mg/kg), as a single dose. Thirty minutes after the administration of solvent or apigenin, LPS (30 μg/kg, i.p.) was given to induce inflammation. All drugs were diluted with 0.9% saline and injected intraperitoneally, a single dose.

**Sampling and cytokines measurement**

Four, twelve, and twenty-four hours after LPS administration, rats were deeply anesthetized by injecting a combination of ketamine and xylazine (50 and 5 mg/kg, i.p.), and then blood samples were taken from the left ventricle. These samples were centrifuged for 15 min at a speed of 4000 rpm. The sera were separated and kept at -80°C. The concentrations of TNF-a, IL-1b, and IL-6 were measured by using Enzyme-linked immune-sorbent assay (ELISA) according to the manufacturer's protocol.

**Statistic method**

Data were analyzed with SPSS Ver.21 software, and using one-way ANOVA and Tukey's tests. P-value less than 0.05 was considered statistically significant.

## Results

The difference between the control group and sham (normal saline) in the measurement intervals was slightly different, but not significant. Therefore, for ease of data presentation, sham results were omitted.

**TNF-a level**

**Figure 1:**
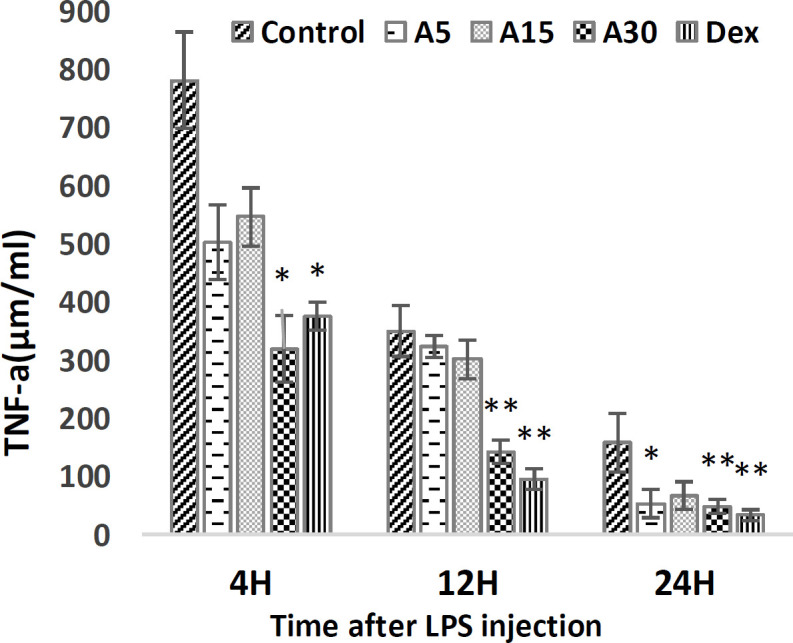
TNF-a level 4, 12, and 24 hr after LPS injection. Pre-injection of 5 (A5), 15 (A15), and 30 (A30) mg/kg of apigenin and 15 mg/kg of dexamethasone (Dex) effect on TNF-a level. The results were normalized with a control group. (*p<0.05 and **p<0.01 compared to control)

Apigenin 30 mg/kg reduced significantly TNF-a levels, 4 (p<0.05), 12 (p<0.01) and 24 (p<0.01) hr after LPS injection, compared to the control group. Also, 24 hr after LPS injection, apigenin 5 and 30 mg/kg significantly decreased TNF-a level, compared to the control (p<0.05 and p<0.01, respectively), but apigenin 15 mg/kg had no significant effect on the level of TNF-a. In the positive control group, dexamethasone significantly reduced TNF-a levels 4 (p<0.05), 12 (p<0.01) and 24 (p<0.01) hr after LPS injection compared to the control group (Figure 1).

**IL-1b level**

**Figure 2:**
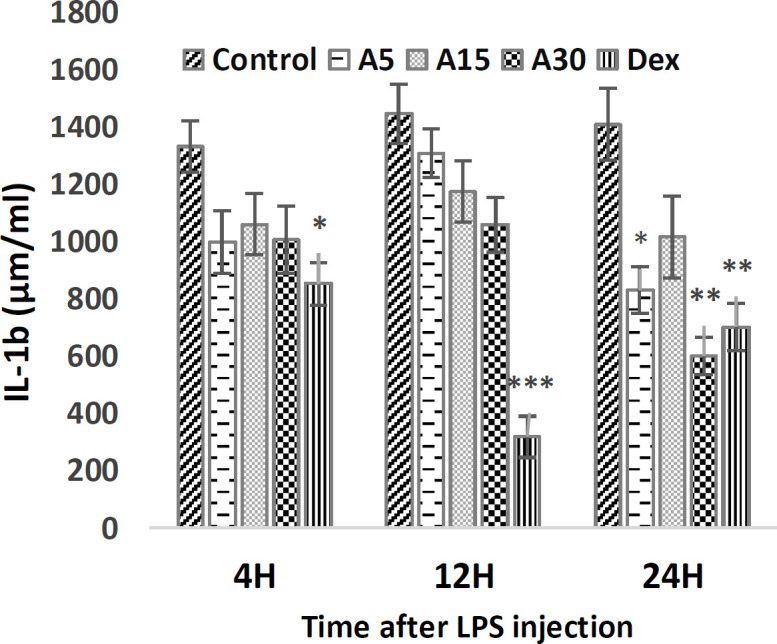
Level of IL-1b 4, 12, and 24 hr after LPS injection. Pre-injection of 5 (A5), 15 (A15) and 30 (A30) mg/kg of apigenin and 15 mg/kg of dexamethasone (Dex) effect on IL-1b level. The results were normalized with a control group. (*p<0.05, **p<0.01, and ***p<0.001 compared to control)

Compared to the control group, apigenin injection at all doses had no significant effect on IL-1b levels, 4 and 12 hr after LPS injection, but 24 hr later, the 5 (p<0.05) and 30 mg/kg (p<0.01) doses of apigenin significantly reduced IL-1b levels. In the positive control group, the level of IL-1b significantly decreased 4 (p<0.05), 12 (p<0.01), and 24 (p<0.01) hr after LPS injection (Figure 2).

**IL-6 level**

**Figure 3:**
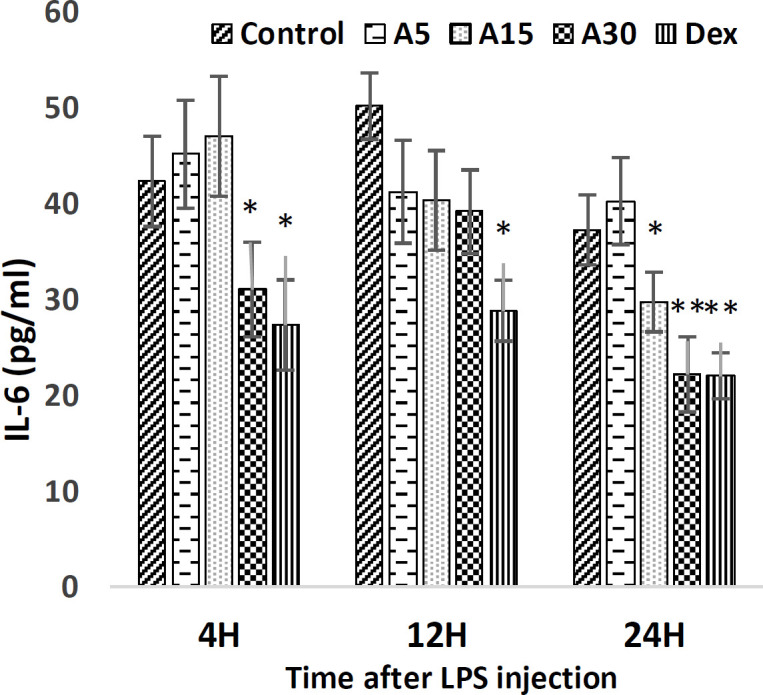
IL-6 level 4, 12, and 24 hr after LPS injection. pre-injection of 5 (A5), 15 (A15), and 30 (A30) mg/kg of apigenin and 15 mg/kg of dexamethasone (Dex) effect on IL-6 level. The results were normalized with a control group. (*p<0.05 and **p<0.01 compared to control)

Within 4 hours after LPS injection, 30 mg/kg of apigenin significantly decreased IL-6 serum level compared to the control (p<0.05). But other doses of apigenin had no significant effect on IL-6 level. Also, apigenin in all three doses had no significant effect on IL-6 level 12 hr after LPS injection. Also, 24 hr after LPS injection, apigenin 15 (p<0.05), and 30 mg/kg (p<0.01) significantly reduced IL-6 levels compared to the control. Dexamethasone also significantly reduced IL-6 levels 4 hr (p<0.05), 12 hr (p<0.05), and 24 hr (p<0.01) after LPS injection in comparison with the control (Figure 3).

## Discussion

This study evaluated the effect of low doses of apigenin on serum levels of pro-inflammatory cytokines in LPS-stimulated rats. The results showed that apigenin decreased the serum level of TNF-a, IL-1b and IL-6, and therefore it can have protective effect against inflammation caused by LPS. According to the results, the suppressive effect of apigenin did not have a dose-dependent pattern. Compared to dexamethasone, the apigenin potency was weak in the initial hours, but 24 hr after the onset of inflammation, apigenin had almost the same effect. The findings of our study are in agreement with the results of some others in this field, for example, it has been reported that in many inflammatory diseases such as sepsis, TNF-a, IL-1b and IL-6 serum levels increase significantly. Therefore, it is recommended to use these changes as a diagnostic marker (Mirzarahimi et al., 2017 ▶; Boskabadi et al., 2013 ▶). TNF-a, IL-1b, and IL-6 regulate different physiological and pathological processes (Mirantes et al., 2014 ▶). On the other hand, the valuable effects of flavonoids in reducing inflammation and oxidative stress are undeniable (Asif, 2012 ▶). These substances are suitable compounds for the management of inflammatory diseases due to their involvement in the intracellular signaling network and related transcription pathways (Suzuki, 2002 ▶). New research evidence has provided the possible use of apigenin as an effective therapeutic agent for various inflammatory diseases (Kowalski et al., 2005 ▶). In a relatively similar study, by examining the effects of apigenin in a murine model of multi-microbial sepsis, it was found that rapid administration of apigenin significantly reduces the construction of pro-inflammatory cytokines and the amount of inflammatory cells (Karamese et al., 2016 ▶). Also, by examining the effects of an apigenin -enriched diet in a Dextran sulfate sodium (DSS) -induced chronic colitis model in rodents, it was shown that apigenin supplementation reduces the symptoms of colitis damage as well as IL-1b and TNF-a levels (Marquez-Flores et al., 2016 ▶). According to another report, the administration of apigenin could reduce the serum levels of TNF-a, IL-1b, IL-6 and the number of foam cells and at the same time reduce the associated inflammation and prevent atherosclerosis (Wang et al., 2015 ▶). One way to create animal models of Parkinsonism (PD) is to use LPS as an endotoxin and produce neuroinflammation. It has been claimed that apigenin (25 and 50 mg/kg) modifies deleterious structural and functional changes through the Nrf2 and NF-κB pathways, and attenuates LPS-induced experimental PD in rats (Patel and Singh, 2022 ▶). 

According to new findings, inflammation can be one of the factors involved in the pathophysiology of depression, and the administration of apigenin (25 and 50 mg/kg) reduces the depressive-related behavior caused by LPS in male rats. The effects of apigenin include suppressing the production of inflammatory cytokines such as IL-1b and TNF-a, and inducing the production of anti-inflammatory cytokines in the frontal cortex (Lee et al., 2015 ▶). In addition, apigenin reduces the translocation of c-Jun translocase to the nucleus and the activation of AP-1 by inhibiting MAPK and ERK (Hu et al., 2016 ▶). 

It has been reported that apigenin (30 μM) significantly down regulates TNF-a, and IL-1b gene expression and reduces their synthesis at the transcriptional level in LPS-activated macrophage. This inhibitory effect could justify the beneficial role of a diet rich in fruits and vegetables in inflammatory diseases (Kowalski et al., 2016 ▶). In this context, by evaluating the effect of 25 μM apigenin concentration on LPS-induced inflammation in intestinal epithelial cells, it was found that apigenin reduced IL-6 gene expression in these cells, but did not affect TNF-a mRNA level (Farkas et al., 2015 ▶). Apigenin has also been reported to significantly reduce the LPS-induced inflammatory response and production of inflammatory cytokines, such as IL-6, IL-1b, and TNF-a, in macrophages through modulation of multiple intracellular signaling pathways, such as prevention of ERK1/2, caspase-1, NLRP3, and NF-kB activation (Zhang et al., 2014 ▶). Another study reported that some apigenin-derived glycosides at non-cytotoxic concentrations inhibited LPS-induced inflammatory responses in humans by reducing serum level of IL-1b and IL-6 (Attiq et al., 2021 ▶). 

In general, apigenin exhibits its anti-inflammatory activities in different ways. According to the results, one of these ways is to reduce the production of TNF-a, IL-1b and IL-6. It seems that, flavonoids can be used in inflammatory diseases specific to humans, and more studies should be done to determine the most suitable dose, time, number, and duration of their administration.

## Conflicts of interest

The authors have declared that there is no conflict of interest.
